# Supplementary material for: Deciphering the Role and Signaling Pathways of PKCα in Luminal A Breast Cancer Cells
Source: Int J Mol Sci. 2022 Nov 14;23(22):14023. doi: 10.3390/ijms232214023 (PMC9696894; doi:10.3390/ijms232214023)
Supplement: Supplementary file 1 [file ijms-23-14023-s001.zip › SFIG.pdf]

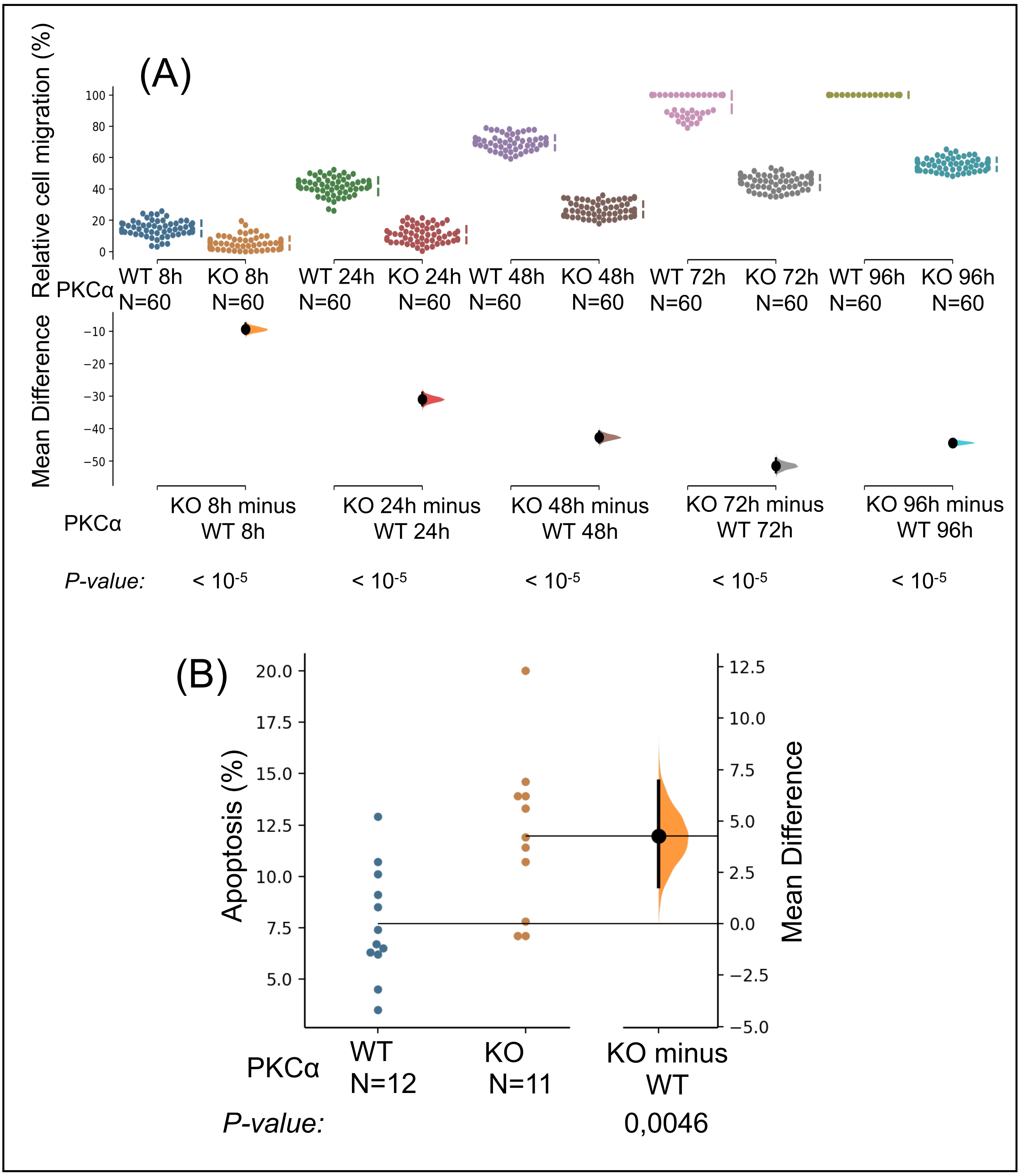

Figure S1. Knock-down of PKCα in MCF-7 cells decreases their migration rate and slightly increases apoptosis. (A) Relative cell migration of MCF-7 PKCα-WT and PKCα-KO cells was measured by wound healing assay for 96 h. The mean difference for comparisons against the PKCα-KO vs PKCα-WT cells are shown in the Cumming estimation plots. The raw data are plotted on the upper axis. On the lower axis, mean differences are plotted as bootstrap sampling distributions (5000). Each mean difference is depicted as a dot. Each 95% confidence interval is indicated by the ends of the vertical error bars. Additional permutation test was also applied to confirm these results, the P values to accept/reject the null hypothesis of no differences between PKCα-WT and PKCα-KO cells are indicated below each comparison (CI 95%). (B) Percentage of apoptosis was measured for the two cell types and the same statistical analysis than (A) was applied, mean difference is plotted on the right size.

(A)

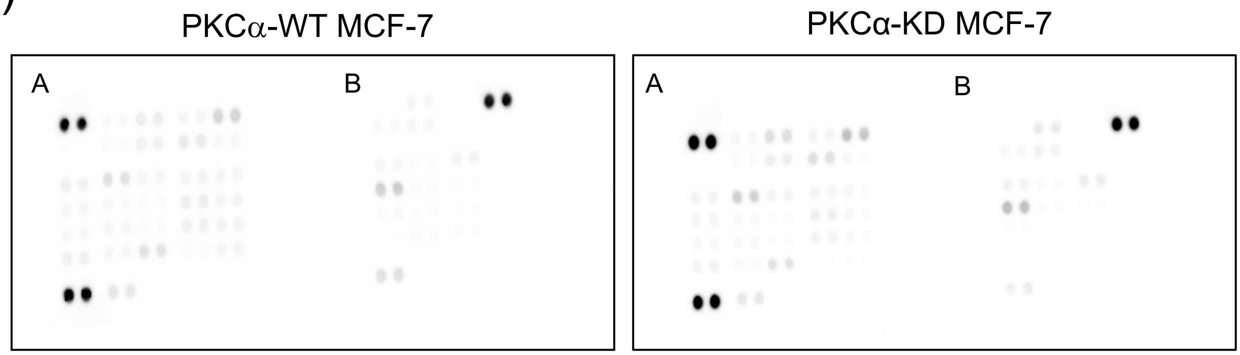

(B)

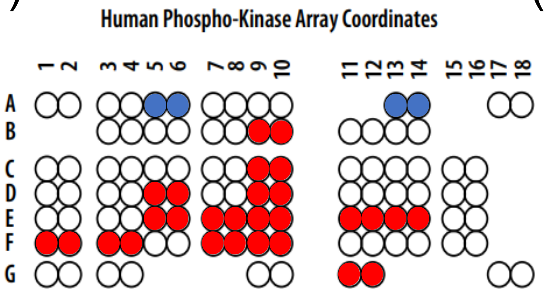

(C)

Target	Phosphorylation Site	Membrane/ Coordinate
p53	S392	B-A13, A14
ERK1/2	T202/Y204 T185/Y187	A-A5, A6
AKT 1/2/3	S473	A-B9, B10
β-catenin	-------	A-C9, C10
Lck	Y394	A-D5, D6
STAT5A	Y694	A-D9, D10
Fgr	Y412	A-E5, E6
STAT6	Y641	A-E7, E8
STAT5B	Y699	A-E9, E10
STAT3	Y705	B-E11, E12
P27	T198	B-E13, E14
HSP60	-------	B-G11, G12
Hck	Y411	A-F1, F2
Chk-2	T68	A-F3, F4
PDGF-rb	Y751	A-F7, F8
STAT5A/B	Y694/Y699	A-F9, F10

(D)

Target	Control	Membrane/ Coordinate
Reference Spot	Positive control	A-G1, G2
PBS	Negative Control	A-G9, G10
Reference Spot	Positive control	B-A17, A18
PBS	Negative Control	B-G17, G18

Figure S2. The relative levels of protein phosphorylation were determined by proteome profiler human phospho-kinase array kit (Cat#: ARY003B, R&D Systems, Minnesota, USA). MCF-7 PKCα-KD were compared versus PKCα-WT cell lysates. (A) WB membranes of the phospho-kinase array. (B) Normalized quantification comparing PKCα-KD (sample) versus PKCα-WT (control). Red dots represent phosphorylated proteins showing a decrease in their phosphorylation levels whereas blue dots are showing an increase. (C) Kinase identification with the phosphorylation sites identified by the Ab used in the kit. D) Each membrane includes its own spots that function as positive control (reference spot) and negative control (PBS). Note that each kinase is tested in two independent spots. Only one biological sample was used in this assay.

Table S1. RT-qPCR validation of microarray data

	GAPDH	SPAM1	PRKCA	ERBB4	PRKA2B	PLCB4	PDGFA	EGFR	CSTA	ITGB6
Ratio	0.994	1.006	0.157	0.720	1.185	0.911	0.859	0.425	0.253	0.454
SD	0.001	0.001	0.006	0.076	0.172	0.076	0.272	0.021	0.012	0.003
CV (%)	0.142	0.141	4.066	10.516	14.506	8.383	31.711	4.991	4.761	0.623
Log2 Ratio	-0.009	0.009	-2.676	-0.475	0.244	-0.134	-0.220	-1.234	-1.986	-1.139
Microarray Log2 ratio		0,06	-3,5	1,6	1,59	1,94	1,74	-2,14	3,34	-3,79

Table S2a.The top eleven gene enrichment functions downregulated after PKCα inhibition.

Description	Term	Log10 p-value	Genes
Interferon Signaling	R-HSA-913531	–31,9779	*BST2, IFI6, GBP1, HLA-A, HLA-B, HLA-C, HLA-F, IFI35, IFIT3, MID1, MT2A, MX1, MX2, OAS1, OAS2, OAS3, PSMB8, STAT1, IFITM1, OASL, UBE2L6, ISG15, TRIM22, IFI30, USP18, TRIM2, SAMHD1, HERC5, XAF1, TRIM5, RSAD2, ANXA1, BCL6, CD36, DUSP6, GRB10, CXCL8, IL15, PIK3R2, PSMB9, SOX2, H3C1, BCL2L11, ITGB6, FOSL1, NMI, IFI44, IFI44L, SHFL, RTP4, ARG2, PPARG, S100A7, S100A8, S100A9, SLPI, KYNU, H2BC11, LY6E, LAMP3, TMPRSS4, ZFP36L1, ZFP36L2, KLF5, EREG, MT1X, PRKCA, SCGB1A1*
Regulation of response to biotic stimulus	GO:0002831	–12,1094	*ARG2, CD36, EREG, HLA-A, HLA-B, HLA-F, IFI35, IL15, OAS1, OAS3, PPARG, PRKCA, STAT1, OASL, NMI, ISG15, USP18, SAMHD1, HERC5, TRIM5, ANXA1, BCL6, ITGA2, MDK, S100A8, S100A9, SCGB1A1, PTGES, BST2, GBP1, GAL, CAV1, H2BC11, TFPI, SEMA3E*
Antiviral mechanism by IFN-stimulated genes	R-HSA-1169410	–10,9912	*MX1, MX2, OAS1, OAS2, OAS3, STAT1, OASL, UBE2L6, ISG15, USP18, HERC5, BID, CXCL8, PIK3R2, PRKCA, TNFSF10, TMPRSS4, RSAD2, EGFR, NMI, ARG2, GBP1, SNAI2, CD36, IFI6*
Negative regulation of cell population proliferation	GO:0008285	–10,2469	*ADM, ARG2, BCL6, CAV1, EREG, IFI35, IFIT3, IGFBP3, CXCL8, IL15, KRT4, PPARG, PLAAT4, SNAI2, SOX2, STAT1, SCGB1A1, FOSL1, H2AC4, IFITM1, PLPP1, NMI, PTGES, IFI30, SULF1, GAL*
Immune response to tuberculosis	WP4197	–9,78627	*IFI35,IFIT3,MX1,OAS1,PSMB8,STAT1,IFITM1,OAS2,OASL,BID,BST2,CTSZ,IFI6, IGFBP3, CXCL8, LGALS3BP, TNFSF10, IFI44L, ISG15, SHFL, XAF1, GBP1, KYNU, NMI, EREG, IL15*
Regulation of peptidase activity	GO:0052547	–9,20569	*BID, BST2, CAV1, IFI6, SERPINB5, PPARG, PSMB8, PSMB9, S100A8, S100A9, SLPI, SOX2, TFPI, TP63, TNFSF10, BCL2L11, TNFAIP8, LAMP3, ANXA8, ADM, ALDH1A3, ANXA1, BCL6, DUSP6, IGFBP3, TGM2, FOSL1, GAL, SIK1, CD36, CTSZ, TRIB2, AURKAIP1, BDNF, SNAI2, EGFR, FTL, STAT1, H2AC4, H2AC11, ITGA2, RGS16, PIK3R2, PRKCA, SIVA1, SGCG*
ER-Phagosome pathway	R-HSA-1236974	–9,10545	*CD36, HLA-A, HLA-B, HLA-C, HLA-F, PSMB8, PSMB9, S100A8, S100A9, TAP2, BID, OAS1, OAS2, OAS3, PIK3R2, STAT1, ISG15, BCL2L11, COL4A5, COL4A6, EGFR, ITGA2, ITGB6, MX1, MX2, OASL, BST2, CAV1, IFI30, PRKCA, SAMHD1, CALML5, TRIM5, ZFP36L1, ZFP36L2, IGFBP3, CXCL8, SGCG, H2AC4, H2BC5, RANBP1, H2BC9, H2BC17, H2BC11, UBE2L6, HERC5, HERC6, COL5A1, BCL6, SLC7A5, RSAD2, IFITM1, BTN3A2, DNM3, TNFSF10, ANXA1, IL15, FOSL1*
Response to wounding	GO:0009611	–8,77802	*ADM, ZFP36L1, ZFP36L2, CD36, COL5A1, ELK3, EREG, ITGA2, ITGB6, KRT6A, MDK, S100A8, SOX2, TFPI, SLC7A11, FLRT3, TMPRSS4, ANXA8, AQP3, CAV1, OAS2, TP63*
Non-genomic actions of 1,25 dihydroxyvitamin D3	WP4341	–8,76795	*CAV1, CYP24A1, CXCL8, OAS2, PRKCA, STAT1, ISG15, IFI44L, RSAD2, IFI6, GBP1, HLA-B, OAS1, PSMB9, ZFP36L1*
Modulation by symbiont of entry into host	GO:0052372	–8,68431	*CAV1, CXCL8, KRT6A, LY6E, IFITM1, TRIM22, TMPRSS4, TRIM5, BST2*
Signaling by Receptor Tyrosine Kinases	R-HSA-9006934	–8,25744	*BDNF, BTC, CAV1, COL4A5, COL5A1, DUSP6, EGFR, EREG, GRB10, ID1, ITGA2, MDK, PIK3R2, PRKCA, STAT1, FOSL1, FLRT3, DNM3, TNS3, BCL2L11, MECOM, PPARG, PSMB8, PSMB9, SNAI2, COL4A6, EIF4B, SLC7A5, ITGB6, SULF1, ANXA1, RANBP1, CDC14B, EPAS1, ERLIN2, LY6E, PSCA, LYPD1, IFI6, TP63, CDK2AP2, TGOLN2, CENPF, AURKAIP1*

Table S2b.The top ten gene enrichment functions upregulated after PKCα inhibition.

Description	Term	Log_{10} p-value	Genes
cellular response to hormone stimulus	GO:0032870	–10,80561172	*CA2, EDN1, EPRS1, ESR1, IGF1R, LYN, ENPP1, PIK3R1, PRKCI, PRLR, PTGER4, SGK1, SMARCC1, STC1, UGCG, NCOA4, NCOA3, SOCS2, NR1D2, NCOA2, DDX17, RAB31, ABHD2, LGR4, HADHA, IGFBP5, TXNIP, ATRX, CDH1, DHX9, MDM2, HSP90B1, EZR, GNA14, ACTR2*
Protein phosphorylation	GO:0006468	–8,673680111	*CDC42, EDN1, EPHA7, ERBB4, IGF1R, LYN, SMAD5, CDK17, PIK3R1, PRKAB2, PRKCI, PRLR, RET, BRD2, SGK1, STK3, DYRK2, CDC42BPA, CPNE3, ULK2, HIPK3, MAP3K2, SMG1, SCYL2, WNK1, HIPK1, EFNB2, GFRA1, PDGFA, SMARCC1, SOCS2, TXNIP, CALM1, PRKAR2B, PTPRO, SH3BP5, CHP1, GPR37, NPY5R, RAP2A, EZR, PJA2, GPNMB, ITCH, RHOB, IGFBP5, SEMA3C, RUFY3, ZNF609, EIF4G1, FXR1*
Regulation of cellular response to stress	GO:0080135	–8,118345022	*CDC42, DHX9, EIF4G1, GPR37, IGF1R, LYN, MDM2, MUC1, PIK3R1, RAP2A, STK3, EZR, FXR1, THOC5, BAG5, BCLAF1, PJA2, ACTR2, HIPK3, AGR2, SERINC3, SMG1, UFL1, OXR1, ITCH*
Intracellular protein transport	GO:0006886	–7,841428472	*TRIM23, RHOB, COPA, HSPA4, KIF5C, KPNA1, MYO6, PIK3R1, PRKCI, RAB6A, STK3, TPR, HSP90B1, NCOA4, USO1, TXNIP, RAB31, CHP1, SNX13, RFTN1, FAF2, SYTL2, SNX16, SNX27*
Regulation of cellular localization	GO:0060341	–7,62597202	*APOD, CALM1, CDC42, CDH1, DHX9, ERBB4, GLUL, LCP1, LMAN1, LYN, PPP1R12A, PIK3R1, PPM1A, PRKCI, TCF7L2, TPR, EZR, PICALM, TCAF1, LYPLA1, AGR2, CHP1, RUFY3, GPD1L, NMD3, PARD6B, UNC13B, RET, NCOA4, MDM2, SOS2, IGF1R, PDGFA*
Pathways in cancer	hsa05200	–7,560140801	*ADCY1, CALM1, RUNX1T1, CDC42, CDH1, EDN1, ESR1, IGF1R, MDM2, PDGFA, PIK3R1, PLCB4, PTGER4, RET, SOS2, TCF7L2, TPR, HSP90B1, NCOA4, NCOA3, GNA13, KCNJ3, NCOA2, PRKCI, PARD6B, DIO1, MAP3K2, KPNA1, UGT2B15, GLUL, SLC38A1, LYN, SOCS2, CANX*
Neuron projection development	GO:0031175	–7,478233099	*ADCY1, ALCAM, APOD, CDC42, CDH1, EDN1, EFNB2, EPHA7, GFRA1, GPR37, IGF1R, KIF5C, LYN, NRCAM, PTPRO, RET, SGK1, PICALM, ULK2, SEMA3C, DICER1, LRP12, PARD6B, PIK3R1, PRKCI, TWF1, RAP2A, RNF6, EZR, FXR1, BAG5, ACTR2, RUFY3, TANC2, OCLN, STC1, CAP2, ATRNL1, RHOB, EIF4G1, SOS2, ST8SIA4, PSME4, PDGFA, PIP5K1A, GPNMB*
RHO gtpase cycle	R-HSA-9012999	–7,348083355	*RHOB, CDC42, LMAN1, MYO6, PCDH7, PIK3R1, TWF1, SOS2, PICALM, CDC42BPA, PKP4, SLC4A7, SRRM1, GNA13, SPEN, FAF2, FAM169A, TOR1AIP1, PARD6B, CALM1, CDH1, PPP1R12A, ACTR2, NCOA2*
Regulation of growth	GO:0040008	–7,224419959	*CDC42, EDN1, EIF4G1, EPHA7, ERBB4, FHL1, IGFBP5, NPY1R, NRCAM, ENPP1, RNF6, SELENOP, SGK1, STK3, EZR, SOCS2, ULK2, SEMA3C, AGR2, RUFY3, RFTN1, LRP12, ITCH, EFNB2, PTPRO, BAG5, CLCN3, RET, APOD, ATRX, PIK3R1, PPM1A, PTGER4, TWF1, RAD21, TPR, PICALM, ID4, DICER1, RUNX1T1, LYN, SPDEF, RC3H2*
Signal transduction by p53 class mediator	GO:0072331	–6,987347799	*ATRX, CASP2, MDM2, MDM4, MUC1, MYO6, DYRK2, USP10, HIPK1, LYN, PIK3R1, RAD21, SGK1, SMG1, SMC5, PSME4, UFL1, WAC, UBE2W, ZBTB38, FHL1, GPNMB, TPR, EIF4G1, RRN3, RHOB, CALM1, ID4, MYBL1, PPP1R12A, CDK17, STAG2, RET, STK3, TCF7L2, BAG5, BCLAF1, SERINC3, CDC42, EDN1, THOC5*

Table S3. Effect of different drugs on proliferation of MCF-7 cells.

DRUG	Time	PKCα-WT MCF-7 DNA fold Change ± SD	% Inhibition Ctrl w/o drug (PKCα-WT MCF-7) vs PKCα-WT MCF-7	PKCα-KO MCF-7 DNA fold Change ± SD	% Inhibition Ctrl w/o drug (PKCα-WT MCF-7) vs PKCα-KO MCF-7
Ctrl w/o drug	Day1	1,01 ± 0,06	------------	1,02 ± 0,05	-0,59
	Day2	1,38 ± 0,09	------------	1,30 ± 0,14	5,80
	Day3	2,07 ± 0,26	------------	1,44 ± 0,29	30,42
	Day4	2,45 ± 0,50	------------	1,55 ± 0,23	36,72
	Day5	2,91 ± 0,61	------------	1,78 ± 0,46	38,98
	Day6	4,29 ± 0,81	------------	2,24 ± 0,66	47,83
	Day7	4,60 ± 1,12	------------	2,65 ± 1,15	42,28
	Day8	5,51 ± 1,04	------------	3,00 ± 1,51	45,52
U73122 1 μM	Day1	0,96 ± 0,04	5,08	0,98 ± 0,07	3,60
	Day2	1,4 ± 0,15	-1,38	1,13 ± 0,06	17,70
	Day3	2,05 ± 0,22	1,26	1,02 ± 0,09	50,87
	Day4	2,56 ± 0,32	-4,24	1,11 ± 0,05	54,92
	Day5	3,26 ± 0,24	-11,94	1,5 ± 0,15	48,45
	Day6	5,08 ± 0,19	-18,55	1,43 ± 0,07	66,57
	Day7	5,61 ± 0,11	-22,05	2,18 ± 0,21	52,59
	Day8	6,41 ± 0,23	-16,32	2,64 ± 0,15	52,05
U73122 10 μM	Day1	0,99 ± 0,1	2,61	1,04 ± 0,14	-2,32
	Day2	1,38 ± 0,12	-0,29	1,17 ± 0,04	14,97
	Day3	1,7 ± 0,09	18,03	1,01 ± 0,06	51,23
	Day4	1,66 ± 0,13	32,27	1,03 ± 0,05	57,87

	Day5	2,08 ± 0,34	28,52	1,04 ± 0,07	64,26
	Day6	2,79 ± 0,34	35,01	0,78 ± 0,05	81,80
	Day7	2,32 ± 0,85	49,55	0,84 ± 0,03	81,79
	Day8	3,49 ± 0,36	36,56	0,83 ± 0,1	84,93
KT5720 0,1 μM	Day1	1,02 ± 0,04	-0,10	1,02 ± 0,08	-0,84
	Day2	1,15 ± 0,07	16,61	1 ± 0,03	27,51
	Day3	2,22 ± 0,14	-7,07	1,12 ± 0,07	45,80
	Day4	2,17 ± 0,1	11,57	0,9 ± 0,07	63,48
	Day5	2,65 ± 0,17	9,11	1,24 ± 0,05	57,47
	Day6	3,66 ± 0,12	14,59	1,19 ± 0,1	72,35
	Day7	4,07 ± 0,17	11,55	1,83 ± 0,05	60,21
	Day8	4,92 ± 0,21	10,74	2,14 ± 0,1	61,14
KT5720 1 μM	Day1	0,98 ± 0,08	3,11	0,96 ± 0,07	5,82
	Day2	1,25 ± 0,05	9,52	1,06 ± 0,05	23,33
	Day3	2,18 ± 0,14	-5,38	1,28 ± 0,06	38,19
	Day4	2,21 ± 0,06	9,73	1,1 ± 0,07	55,32
	Day5	2,69 ± 0,12	7,73	1,27 ± 0,06	56,27
	Day6	3,53 ± 0,15	17,56	1,37 ± 0,09	68,14
	Day7	3,91 ± 0,07	14,92	1,87 ± 0,18	59,28
	Day8	4,55 ± 0,19	17,46	2,01 ± 0,12	63,50
BMS 599626 0,1 μM	Day1	0,93 ± 0,04	8,53	0,94 ± 0,1	7,79
	Day2	1,31 ± 0,09	5,16	0,96 ± 0,21	30,41
	Day3	1,73 ± 0,27	16,47	1,35 ± 0,17	34,81

	Day4	2,2 ± 0,22	10,34	1,4 ± 0,05	42,88
	Day5	2,2 ± 0,06	24,40	1,67 ± 0,21	42,78
	Day6	2,47 ± 0,18	42,30	2,01 ± 0,18	53,21
	Day7	2,54 ± 0,14	44,82	1,96 ± 0,31	57,38
	Day8	2,49 ± 0,2	54,78	2,03 ± 0,3	63,09
BMS 599626 1 μM	Day1	0,92 ± 0,28	9,02	0,99 ± 0,14	2,37
	Day2	1,24 ± 0,22	9,88	1,08 ± 0,13	21,69
	Day3	1,72 ± 0,35	16,95	1,27 ± 0,36	38,92
	Day4	2,26 ± 0,29	7,89	1,38 ± 0,04	43,70
	Day5	2,35 ± 0,29	19,42	1,36 ± 0,23	53,35
	Day6	2,52 ± 0,09	41,31	1,75 ± 0,4	59,28
	Day7	2,31 ± 0,12	49,71	1,95 ± 0,13	57,70
	Day8	2,45 ± 0,27	55,55	1,99 ± 0,21	63,95
BMS 599626 5 μM	Day1	0,99 ± 0,05	2,37	0,96 ± 0,04	5,08
	Day2	1,26 ± 0,08	8,43	1,08 ± 0,04	21,88
	Day3	1,6 ± 0,11	22,98	1,32 ± 0,09	36,14
	Day4	1,51 ± 0,18	38,39	1,24 ± 0,24	49,41
	Day5	1,73 ± 0,07	40,55	1,34 ± 0,09	54,12
	Day6	1,64 ± 0,09	61,84	1,12 ± 0,22	73,92
	Day7	2,05 ± 0,11	55,48	1,49 ± 0,15	67,60
	Day8	2,01 ± 0,07	63,59	1,57 ± 0,34	71,49

BMS 599626 10 μM	Day1	1 ± 0,08	1,63	1,05 ± 0,05	-3,80
	Day2	1,16 ± 0,1	15,70	1,11 ± 0,06	19,15
	Day3	1,34 ± 0,17	35,42	1,19 ± 0,14	42,42
	Day4	1,22 ± 0,15	50,12	0,92 ± 0,31	62,57
	Day5	1,24 ± 0,08	57,47	1,13 ± 0,05	61,08
	Day6	1,04 ± 0,06	75,79	0,79 ± 0,2	81,51
	Day7	1,11 ± 0,19	75,97	0,66 ± 0,12	85,70
	Day8	0,99 ± 0,34	82,08	0,41 ± 0,13	92,55
Imatinib 1 μM	Day1	1,01 ± 0,04	0,15	0,98 ± 0,07	3,60
	Day2	1,22 ± 0,07	11,16	1,13 ± 0,06	17,70
	Day3	1,51 ± 0,07	27,09	1,02 ± 0,09	50,87
	Day4	1,77 ± 0,11	27,68	1,11 ± 0,05	54,92
	Day5	2,82 ± 0,06	3,26	1,5 ± 0,15	48,45
	Day6	2,71 ± 0,07	36,81	1,43 ± 0,07	66,57
	Day7	3,68 ± 0,26	20,08	2,18 ± 0,21	52,59
	Day8	3,24 ± 0,06	41,20	2,64 ± 0,15	52,05
Imatinib 10 μM	Day1	1,03 ± 0,03	-1,58	1,04 ± 0,14	-2,32
	Day2	1,16 ± 0,08	15,88	1,17 ± 0,04	14,97
	Day3	1,25 ± 0,03	39,52	1,01 ± 0,06	51,23
	Day4	1,46 ± 0,05	40,43	1,03 ± 0,05	57,87
	Day5	2 ± 0,14	31,44	1,04 ± 0,07	64,26
	Day6	2,01 ± 0,09	53,21	0,78 ± 0,05	81,80
	Day7	2,55 ± 0,06	44,49	0,84 ± 0,03	81,79
	Day8	2,94 ± 0,28	46,59	0,83 ± 0,10	84,98

Table S4. Effect of different drugs on migration of MCF-7 cells.

DRUG	Time	PKCα-WT MCF-7 migration (%) ± SD	% Inhibition vs Ctrl w/o drug (PKCα-WT MCF-7) on PKCα-KO MCF-7	PKCα-KO MCF-7 migration (%) ± SD	% Inhibition vs Ctrl w/o drug (PKCα-WT MCF-7) on PKCα-KO MCF-7
Ctrl w/o drug	8h	14,08 ± 5,04	------------	5,46 ± 4,20	63,16
	24h	41,76 ± 5,62	------------	10,84 ± 5,34	74,05
	48h	69,59 ± 5,04	------------	26,99 ± 4,90	61,21
	72h	95,44 ± 6,78	------------	44,06 ± 4,77	53,84
U73122 1 μM	8h	8,51 ± 4,58	42,60	0,47 ± 5,09	96,76
	24h	31,29 ± 6,44	25,07	7,89 ± 7,16	81,08
	48h	54,44 ± 7,11	21,77	14,14 ± 13,72	79,67
	72h	80,56 ± 14,17	15,59	22,53 ± 10,02	76,39
U73122 10 μM	8h	4,18 ± 3,08	71,79	0,54 ± 2,12	96,35
	24h	16,36 ± 9,17	60,82	-0,28 ± 4,33	100,66
	48h	21,35 ± 6,13	69,32	6,38 ± 3,57	90,83
	72h	44,85 ± 9,46	53,01	10,50 ± 7,16	89,00
KT5720 0,1 μM	8h	15,96 ± 4,73	-7,70	1,99 ± 3,72	86,55
	24h	44,37 ± 8,03	-6,25	5,91 ± 2,92	85,84
	48h	58,41 ± 5,64	16,06	6,29 ± 3,05	90,96
	72h	94,57 ± 7,92	0,92	12,57 ± 5,62	86,83
KT5720 1 μM	8h	7,16 ± 4,7	51,69	3,62 ± 2,65	75,57
	24h	27,17 ± 5,94	34,95	4,59 ± 2,97	89,01

	48h	42,71 ± 6,46	38,63	6,54 ± 3,86	90,60
	72h	65,65 ± 5,69	31,21	11,97 ± 4,13	87,46
BMS 599626 0,1 μM	8h	10,41 ± 3,55	29,78	3,37 ± 2,90	77,29
	24h	29,94 ± 3,65	28,32	7,22 ± 5,24	82,72
	48h	54,75 ± 5,58	21,32	13,43 ± 4,02	80,70
	72h	71,66 ± 4,64	24,92	22,03 ± 5,18	76,91
BMS 599626 1 μM	8h	9,00 ± 3,99	39,26	3,20 ± 3,06	78,44
	24h	25,18 ± 4,67	39,71	7,06 ± 3,78	83,09
	48h	49,91 ± 4,92	28,27	16,63 ± 4,71	76,10
	72h	63,69 ± 3,74	33,27	18,56 ± 5,24	80,56
Imatinib 1 μM	8h	15,64 ± 4,38	-5,54	5,44 ± 2,95	63,32
	24h	48,90 ± 4,61	-17,10	19,82 ± 4,14	52,53
	48h	81,49 ± 3,05	-17,11	37,87 ± 5,98	45,58
	72h	94,99 ± 7,45	0,47	56,72 ± 5,19	40,57
Imatinib 10 μM	8h	15,70 ± 4,67	-5,90	3,63 ± 2,48	75,51
	24h	48,92 ± 4,45	-17,14	11,83 ± 4,18	71,68
	48h	68,01 ± 4,36	2,26	22,28 ± 4,48	67,98
	72h	82,01 ± 4,13	14,07	29,76 ± 4,33	68,82

Table S5. Effect of different drugs on apoptosis of MCF-7 cells.

DRUG	PKCα-WT MCF-7 Effect (%) ± SD	Effect vs Ctrl no drug (PKCα-WT MCF-7) on PKCα-WT MCF-7	PKCα-KO MCF-7 Effect (%) ± SD	Effect vs Ctrl no drug (PKCα-WT MCF-7) on PKCα-KO MCF-7
Ctrl no drug	7,7 ± 2,68	------------	11,97 ± 3,84	155,49
U73122 1 μM	12,47 ± 5,31	161,90	13,2 ± 4,04	171,43
U73122 10 μM	26,10 ± 4,55	338,96	22,47 ± 6,42	291,77
U73122 20 μM*	22,96 ± N.D	298,18	44,18 ± N.D	573,77
KT5720 0,1 μM	7,87 ± 1,61	102,16	10,00 ± 2,23	129,87
KT5720 1 μM	7,57 ± 1,90	98,27	8,63 ± 1,62	112,12
KT5720 10 μM	13,11 ± 0,53	170,22	14,94 ± 0,38	194,03
BMS 599626 0,1 μM	9,87 ± 1,24	128,14	13,07 ± 0,31	169,70
BMS 599626 1 μM	11,70 ± 1,61	151,95	14,37 ± 0,65	186,58
BMS 599626 20 μM*	33,69 ± N.D	437,53	49,17 ± N.D	638,57
Imatinib 1 μM	18,73 ± 5,90	243,29	18,30 ± 3,01	237,66
Imatinib 10 μM	28,90 ± 5,74	375,32	24,07 ± 2,35	312,55
Imatinib 20 μM*	24,49 ± N.D	318,05	26,88 ± N.D	349,09

*These experiments have been only performed with one biological sample.

Table S7. Oligonucleotide sequences used in qPCR assays.

RefSeq	Gene name	Oligonucleotide sequence	Concentration (nM)
NM_002046	GAPDH	5’ ACATCGCTCAGACACCATG<br>3’ TGTAGTTGAGGTCAATGAAGGG	100
NM_002737	PRKCA	5’TCCAGATTATATCGCCCCAGA<br>3’ CCGGCAAGCATTTCATACAAC	100
NM_001042599	ERBB4	5’CAGATGCTACGGACCTTACG<br>3’ AACACATGCTCCACTGTCAT	100
NM_001172646	PLCB4	5’AGATCGAAAGTGCTGACCAAG<br>3’ GCTCATCTTCTACAGTGACCAG	100
NM_002736	PRKA2B	5’CTACAATCACTGCTACCTCTCC<br>3’ TCTCTTTTTGGCATTGTTTTTCAC	100
NM_005228	EGFR	5’CTGCTGCCACAACCAGT<br>3’ TTCACATCCATCTGGTACGTG	200
NM_000888	ITGB6	5’TGGTGACCCCTGTAACTCTA<br>3’ AGAACCATCCTTTGAGAAATCTTC	100
NM_033023	PDGFA	5’CGTCCGCCAACTTCCTG<br>3’ CTTCCTGACGTATTCCACCCTT	200
NM_001174046	SPAM1	5’GAGACATTGGGTGGCTGGAT<br>3’ AGGATTGTTTTTCAAGTTGCATCA	200
NM_005213	CSTA	5’ACTAGTCAAGCCTAATGCAACT<br>3’ GTGAATTGATAGTCCACGATCCA	200
